# Recall of Autobiographical Memories Following Odor vs Verbal Cues Among Adults With Major Depressive Disorder

**DOI:** 10.1001/jamanetworkopen.2023.55958

**Published:** 2024-02-13

**Authors:** Emily K. Leiker, Emma Riley, Scott Barb, Sair K. Lazzaro, Laurie Compère, Carolyn Webb, Gia Canovali, Kymberly D. Young

**Affiliations:** 1 Department of Psychiatry, University of Pittsburgh, Pittsburgh, Pennsylvania; 2 University of Pittsburgh Medical Center, Pittsburgh, Pennsylvania

## Abstract

**Importance:**

Major depressive disorder (MDD) is associated with deficits in autobiographical memory (AM) recall, which is thought to stem from disruptions in effortful recall. Understanding whether these deficits are mitigated when recall is stimulated more directly, such as by odor cues, could inform therapeutic interventions for MDD.

**Objective:**

To evaluate whether deficits in specific AM recall in MDD are mitigated when odor cues vs word cues are used to prompt memory.

**Design, Setting, and Participants:**

This cross-sectional study assessed recall of specific AMs in response to both odor cues and word cues (in a randomized, counterbalanced order) in a repeated measures design. Data were collected between September 2021 and November 2022. The study took place at the University of Pittsburgh School of Medicine in Pennsylvania and included adults with a primary diagnosis of MDD, according to the Mini International Neuropsychiatric Interview. Data were analyzed from January to June 2023.

**Main Outcomes and Measures:**

The primary outcome measure was the percentage of specific AMs recalled in response to odor-cued memories vs word-cued memories. Additional outcome measures included ratings of arousal, vividness, repetition, and recall response time for odor-cued memories vs word-cued memories.

**Results:**

Thirty-two adults (mean [SD] age, 30.0 [10.1] years; 26 [81.3%] female; 6 [18.8%] male) with a primary diagnosis of MDD completed the study. Participants recalled more specific AMs for odor cues than word cues (mean [SD], 68.4% [20.4%] vs 52.1% [23.3%]; Cohen* d*, 0.78; *P* < .001). Additionally, odor-cued recall was rated more arousing (mean [SD], 3.0 [0.8] vs 2.6 [0.7]; Cohen* d*, 1.28; *P* < .001) and vivid (mean [SD], 3.3 [0.7] vs 3.0 [0.7]; Cohen* d*, 0.67; *P* < .001), and was slower than word-cued recall (mean [SD], 14.5 [3.6] vs 8.9 [3.4] seconds; Cohen* d*, 1.18; *P* < .001). When compared with the population mean for word cues in healthy controls (80%), participants recalled fewer specific memories in response to words (Cohen* d*, 1.18; *P* < .001), supporting the presence of overgenerality. Notably, the percentage of specific memories recalled in response to odor cues did not differ from the healthy control population mean (Cohen* d*, 0.26; *P* = .15).

**Conclusions and Relevance:**

In this cross-sectional study, adults with MDD recalled more specific AMs in response to odor cues compared with word cues. This study suggests that AM deficits may only be observed when verbal cues are used and provides a potential new method for increasing specific AM recall in patients with MDD.

## Key Points

**Question:**

Is the recall of specific autobiographical memories (AMs) improved in individuals with major depressive disorder (MDD) when prompted using odor cues vs word cues?

**Findings:**

In this cross-sectional study of 32 adults with MDD, odor cues were associated with more specific AM recall compared with word cues.

**Meaning:**

These findings suggest that odor cues may mitigate AM recall deficits in MDD compared with verbal cues, highlighting a new potential avenue for strengthening specific AM recall in depression.

## Teaser

This cross-sectional study examines specific autobiographical memory recall among US adults with major depressive disorder when odor cues vs memory cues are used to prompt memory.

## Introduction

Major depressive disorder (MDD) is a pervasive and common illness that severely limits and diminishes individuals’ quality of life.^1^ Deficits recalling specific autobiographical memories (AMs) represent a cognitive feature associated with MDD, with patients reporting fewer specific and more categorical memories when presented with emotionally valanced cue words, relative to healthy controls.^2^ A specific memory refers to a memory for a single event that occurred at an identified place within a 24-hour period, whereas a categorical memory refers to a generalized memory for a category of events encompassing several distinct episodes rather than 1 specific event.^2^ This cognitive deficit persists despite the remission of depressive symptoms, raising the possibility that overgeneral AM recall may reflect a trait-like marker of MDD^3,4^ with a potentially causal role in the development of this disorder.^3,5^

AM is believed to be organized hierarchically, where abstract descriptions of general self-knowledge lead to the recall of specific episodic memories.^6^ Theories of overgeneral AM in MDD suggest patients get stuck in a conceptually based retrieval search, biasing recall toward overgeneralized self-knowledge statements like “I am a failure” or “I fight with my friends a lot,” and reducing access to specific event memories.^7^ Notably, studies examining AM overgenerality in patients with MDD have all used words or pictures to cue memory retrieval.^8^ Such modalities may favor a verbally mediated hierarchical search process over direct episodic recall. Odor cues, by contrast, have been found to elicit AM recall in healthy participants even when the odor involved cannot be verbally identified, suggesting that odor cues may facilitate a more direct and less verbally mediated retrieval process.^9^ To our knowledge, no study has examined whether the overgeneral AM phenomenon in MDD exists when odor cues, which may trigger specific episodic recall more directly, are used.

Memories triggered by odors typically differ from memories triggered by words in several recall characteristics.^10^ Odor cues are consistently found to evoke memories that are more emotionally arousing and associated with stronger feelings of reliving than memories evoked by words.^10,11^ This characteristic is referred to as the Proust phenomenon, which ascribes odors the ability to cue highly vivid AMs.^12^ However, the recall characteristics of odor-cued AMs have yet to be studied in a patient population, such as patients with MDD, which this study aims to investigate.

Odor-evoked memories may be unique relative to other stimuli, such as auditory and visual stimuli, and contain such emotionality due to the unique neuroanatomy of olfactory processing in the brain. The olfactory bulb is responsible for processing odor information critical for individuals’ assessments of their environment.^13^ The olfactory bulb directly projects to several structures in the brain implicated in memory and emotion, including the amygdala and hippocampus, allowing olfactory information to enter the limbic system directly (instead of relayed through the thalamus like other sensory information). Some have suggested that the efficacy of odor cues for retrieving rich AMs is due in part to the link between arousal and affective reactions mediated by the amygdala.^14^ Indeed, relative to verbal cues, odor cues engage a more direct retrieval route, evoking more activity in medial temporal regions, such as the hippocampus and amygdala, compared with the widespread prefrontal activity elicited for verbally cued memories.^15,16^ Thus, despite some suggestions that patients with MDD show reduced olfactory sensitivity and olfactory bulb volume,^17^ our primary interest in this investigation is odor-cued activation of other limbic regions, such as the amygdala in this population, rather than deficits in the olfactory bulb itself. Given that patients with MDD tend to have prefrontal dysfunction,^18^ we hypothesize the direct link of olfaction to limbic regions will result in more specific AMs recalled for odor cues than verbal cues.

We have recently found that increasing amygdala activity during positive AM recall in patients with MDD increases their ability to recall specific AMs.^19^ This suggests that the amygdala may be a core component underlying the AM deficit in MDD. By using odor cues that will directly stimulate the amygdala and the hippocampus, we hypothesize that the overgenerality bias typically found in MDD will be reduced.

## Methods

This cross-sectional study followed the Strengthening the Reporting of Observational Studies in Epidemiology (STROBE) reporting guideline.^20^ The research protocol was approved by the University of Pittsburgh institutional review board. Participants gave written informed consent to participate in the study after receiving a complete description, and they received financial compensation.

### Participants

Participants included 32 individuals aged 18 to 55 years with a primary diagnosis of MDD. Participants were recruited from the community via online advertisements. Diagnostic screening evaluations were performed virtually by the study clinician using secure video conferencing software. The Beck Depression Inventory-II (BDI-II)^21^ and Mini International Neuropsychiatric Interview (MINI)^22^ were administered virtually to participants on the day of the study visit. Exclusion criteria included current or history of psychosis; bipolar I or bipolar II; major medical or neurological disorders; drug or alcohol abuse within the previous year or lifetime alcohol or drug dependence (expect nicotine); and current allergies, cold, COVID-19, or other medical issues altering the sense of smell. Current antidepressant medications were not an exclusion, but participants had to be stable on their medication, which was defined as maintaining the same dose for at least 3 weeks or 6 weeks for fluoxetine. Data were collected between September 2021 and November 2022. Demographic information regarding race, ethnicity, and sex was assessed via participant self-report based on classifications from the PhenX toolkit.^23^ Race and ethnicity were assessed to determine the representativeness and generalizability of our sample.

### Autobiographical Memory Task

Participants were presented with a series of cues and asked to recall a specific memory from their life in response to each cue. Prior to the task, participants were instructed and presented with the following example of a specific memory: “If the word bold made you think of the fact that you tend to go to your local coffee shop every morning that would not be a specific memory because it’s not a single event. Instead, you would want to think of a particular morning that you went to your local coffee shop.” The response time (RT) to recall a memory for each cue, defined as the latency from cue presentation to the first word of each response,^19^ was recorded by the experimenter with a stopwatch. If a participant did not recall a memory within 30 seconds of being presented a cue or began to repeat a memory previously recalled for a different cue, it was recorded as a no memory trial. The experimenter recorded participant memories verbatim to allow for later coding of memory specificity.

### Materials

A total of 24 items that could be presented as either odor or word cues were selected from Chu et al^24^ (Table 1). For each participant, half the cues were presented as odors and the other half as words in separate blocks. Block order and cue modality were counterbalanced between participants, with half of participants receiving odor cues first (and vice versa) and each cue presented as either a word or odor evenly across participants. Odor samples of each stimulus were stored and presented in small opaque glass jars with screw-top airtight lids and were regularly replaced to maintain freshness. Depending on viscosity of the stimulus, some were placed on cotton balls within the jars to maintain a concentrated odor. In the word condition, the labels for each odor were verbally presented by the experimenter.

**Table 1.** Odor Cue Substances and Corresponding Word Cues^a^

Odor cue	Word cue
Clove bulbs	Clove
Orange essential oil	Orange
Tomato ketchup	Tomato
Ground coffee	Coffee
Apple cider vinegar	Vinegar
Dried herbal mint	Mint
Coconut oil	Coconut
Mustard powder	Mustard
Scotch whiskey	Whiskey
Dried herbal oregano	Oregano
Dried cumin powder	Curry
Red wine	Wine
Vanilla extract	Vanilla
Tobacco ash	Cigarette
Cough syrup	Medicine
Lavender essential oil	Lavender
Liquid hand soap	Soap
Liquid disinfectant	Disinfectant
Black liquid ink	Ink
Wax shoe polish	Shoe polish
Vicks Vaporub	Menthol
Rose essential oil	Rose
Cinnamon powder	Cinnamon
Cheese powder	Cheese

^a^ Cues were adapted from odor substances and labels from the PhenX ToolKit.^23^

Memories were coded by the experimenter according to their level of specificity using conventional definitions for coding AMs.^24^ Depression severity was calculated using scores from the BDI-II.^21^ The cutoff score ranges are as follows: 0 to 13, minimal; 14 to 19, mild; 20 to 28, moderate; and 29 to 63, severe. Minimal scores were not observed because the study included only individuals with a current diagnosis of MDD.

### Procedure

Participants were asked to refrain from smoking for at least 1 hour before the experiment and to avoid wearing any perfumes or colognes to the experiment. They were also asked not to eat any strong-smelling foods at least 2 hours prior to the experiment. In this within-participants design, participants were presented with 12 odors and 12 words and asked to recall a memory from their lives for each cue. When presented with odors, participants were instructed to keep their eyes closed when smelling the odor to eliminate any visual biases. The experimenter recorded then memories verbatim and then later coded for memory specificity. Participants were asked to rate each of their retrieved memories on valence (positive, negative, no memory), arousal (5-point scale from very low to very high), vividness (5-point scale from not at all vivid to perfectly clear and vivid), and repetition (“How often do you think about this memory?” rated on a 5-point scale with the options: never thought of it until now, rarely [1 to 5 times], sometimes [6 to 25 times], often [25 to 100 times], or all the time [100 times or more]). The ratings were obtained directly following memory recall, and participants were prompted by the experimenter to give their rating before proceeding to the next cue. Participants were also asked to identify each odor presented to them to assess for any influence of odor recognition accuracy but were reminded that correct identification was not essential to the experiment.

### Statistical Analysis

All statistical analyses were performed in SPSS Statistics version 28 (IBM). We conducted repeated measures analysis of the variance (ANOVAs) to examine within-participants associations of cue type (odors or words) on the following dependent variables: mean percentage of memories recalled with each specificity level (specific, categorical, extended, semantic, none) and valence (positive, negative). A third repeated measures ANOVA examined the associations of cue type on the properties of the recalled memories (ie, arousal, vividness, repetition, RT). Significant within-participant tests were followed up using 2-tailed paired sample *t* tests for the relevant post hoc pairwise comparisons. Alpha level was set to .05 for all analyses, with Bonferroni correction (*P* ≤.005) applied to all follow-up *t* tests to correct for multiple comparisons. Data were collected from September 2021 to November 2022 and analyzed from January to June 2023.

## Results

This study included 32 adults with MDD (mean [SD] age, 30.0 [10.1] years; 26 females [81.3%]; 6 males [18.8%]; 4 Black participants [12.5%]; 7 Asian Indian participants [21.8%]; and 21 White participants [65.6%]). Additional demographic information regarding the sample is shown in Table 2. Odors were correctly identified a mean (SD) of 29% (16%) of the time. For the specificity repeated measures ANOVA, there was a significant cue-by-specificity level interaction (mean [SD], 68.4% [20.4%] vs 52.1% [23.3%]; *F*_4,28_ = 9.60; *η_p_^2^* = 0.23; *P* < .001) (Figure). Follow-up paired *t* tests revealed that odor cues resulted in more specific memories (*t*_31_ = 4.43; Cohen* d*, 0.78; *P* < .001) relative to word cues, while word cues resulted in more categorical (*t*_31_ = 3.00; Cohen* d*, 0.49; *P* = .005) and no memory responses (*t*_31_ = 3.84; Cohen* d*, 0.68; *P* < .001) relative to odor cues. Differences between extended and semantic memories failed to reach statistical significance (*t*_31_ < 2.16; Cohen* d*, <0.39; *P* = .12).

**Table 2.** Demographic and Clinical Characteristics Among 32 Participants With Major Depressive Disorder

Characteristic	Participants, No. (%)
Age, mean (SD) range, y	30 (10.1) [20-52]
BDI-II score, mean (SD) range^a^	27.2 (7.1) [15.0-43.0]
Race and ethnicity^b^	
Asian Indian	7 (21.8)
Black	4 (12.5)
Chamorro	0
Chinese	0
Filipino	0
Japanese	0
Korean	0
Native Hawaiian	0
Samoan	0
Vietnamese	0
White	21 (65.6)
Other^c^	0
Sex^b^	
Male	6 (18.8)
Female	26 (81.3)

Abbreviations: BDI-II, Beck Depression Inventory-II.

^a^ Depression symptoms were assessed via participant self-report on the BDI-II,^20^ where symptom severity cutoff scores are as follows: minimal, 0 to 13; mild, 14 to 19; moderate, 20 to 28; and severe, 29 to 63. All participants met criteria for current depression (assessed with the Mini International Neuropsychiatric Interview)^21^ as a condition of enrollment.

^b^ Ethnicity and sex assigned at birth were assessed via participant self-report based on the PhenX toolkit.^23^

^c^ All other races and ethnicities not listed.

**Figure.**
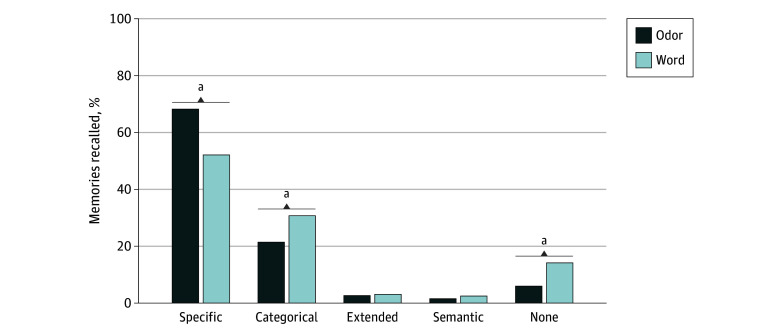
Percentage of Memories Recalled When Comparing Memory Specificity Level for Each Cue Type ^a^*P* for Bonferroni correction ≤.005.

For the valence repeated measures ANOVA, there was a cue-by-valence interaction (*F*_1,30_ = 4.46; *η_p_^2^* = 0.13; *P* = .04). While participants nominally recalled more positive and fewer negative memories for odor cues than word cues, this difference did not survive correction for multiple comparisons (*t*_31_ < 2.16; Cohen* d*, <0.38; *P* >.038).

The final repeated measures ANOVA examined the associations of cue type with the properties of recalled memories. There was a main association of cue type on arousal (*F*_1,31_ = 52.28; *η_p_^2^* = 0.63; *P* < .001), vividness (*F*_1,31_ = 14.34, *η_p_^2^* = 0.32; *P* < .001), and RT (*F*_1,31_ = 44.86; *η_p_^2^* = 0.59; *P* < .001) but not repetition (*F*_1,31_ = 3.58; *η_p_^2^* = 0.10; *P* = .07). Odor-cued memories were significantly more arousing (mean [SD], 3.0 [0.8] vs 2.6 [0.7]; *t*_31_ = 7.23; Cohen* d* = 1.28; *P* < .001), vivid (mean [SD], 3.3 [0.7] vs 3.0 [0.7]; *t*_31_ = 3.79; Cohen* d* = 0.67; *P* = .001), and slower to recall (mean [SD], 14.5 [3.6] vs 8.9 [3.4] seconds; *t*_31_ = 6.70; Cohen* d* = 1.18; *P* < .001) than word-cued memories (Table 3). We also assessed the association of cue presentation order on specific or positive memory retrieval, comparing participants who encountered odor vs word cue presentations first. Independent sample *t* tests revealed no significant associations of cue presentation order on either the number of specific memories or positive memories recalled. No variable was associated with BDI-II score.

**Table 3.** Autobiographical Memory Characteristics for Odor Cues vs Word Cues

Characteristic	Participants, mean (SD)		Odor cues vs word cues		
	Odor cues	Word cues	*t*_31_	*P* value^a^	Cohen *d*
% of Memories recalled, by specificity					
Specific	68.4 (20.4)	52.1 (23.3)	4.43	<001^b^	0.78
Categorical	21.4 (15.3)	30.7 (19.9)	−3.00	.005^b^	0.49
Semantic	1.6 (3.3)	0.8 (2.5)	1.00	.33	0.18
Extended	2.7 (3.3)	3.1 (6.3)	−1.18	.25	0.21
No memory	6.0 (8.0)	14.1 (11.7)	−3.84	<.001^b^	0.68
% of Memories recalled, by valence					
Positive	71.6 (15.6)	63.1 (16.9)	2.16	.04	0.38
Negative	28.4 (16.2)	36.9 (17.1)	−2.05	.05	0.37
Recall speed and ratings					
Response time, s	14.5 (3.6)	8.9 (3.4)	6.70	<.001^b^	1.18
Arousal	3.0 (0.8)	2.6 (0.7)	7.23	<.001^b^	1.28
Vividness	3.3 (0.7)	3.0 (0.7)	3.79	<.001^b^	0.67
Repetition	2.1 (0.4)	2.2 (0.6)	−1.90	.07	0.33

^a^ *P* for Bonferroni-corrected *P* values. Statistical significance was set at *P* ≤.005.

^b^ Statistically significant.

### Power Considerations

For a within-participants *t* test with a 2-tailed *P* = .05 and an observed effect size of 0.78 for our main hypothesis regarding specific AM recall, we had an observed power of 0.99 with our sample size 32 participants. However, for the smaller effect sizes observed (d = 0.38) for other properties of the recalled memories, such as valence, our observed power for a 2-tailed *P* < .05 was only 0.55 and a sample size of 90 participants would be needed to achieve 0.80 power.

## Discussion

While much research has been done to examine the properties of odor-cued memories in healthy participants, to our knowledge, this is the first study to examine AM recall using odor cues in participants with MDD. Participants with MDD recalled more specific AMs when cued with odors than with words, which supports our primary hypothesis. This increased specificity occurred despite participants’ difficulty identifying the odors presented. Indeed, odors were correctly identified only 29% of the time, similar to previous findings of odor recognition accuracy of approximately 30% in participants with MDD.^25^ This suggests that whether an individual is able to accurately identify an odor is independent from its ability to trigger AM recall. Despite relatively poor odor recognition performance, odor cues in our study elicited such high rates of AM recall that it supports the hypothesis that odor cues may activate a direct route to recall rather than a verbally mediated one. This may represent a unique strength of odor cues, making them better suited for translation to improving memory in depression than word cues, given that word cues are subject to constraints stemming from verbally and prefrontally mediated processing deficits in depression.

Odor-cued memories were rated overall as more vivid and more arousing than memories cued with words, consistent with findings in previous studies using healthy controls.^10,11^ We also note that no order association was found using this within-participant design, meaning the percentage of memories recalled for both specific and positive memories did not differ based on whether the participant was presented odor or word cues first. Furthermore, stimuli used as odors and words were counterbalanced. Thus, the potential confounds of presentation order and differences in properties, such as arousal or frequency of use, for the 2 cue modalities were controlled.

We did not find an association between memory performance and depression severity as measured using the BDI. This is consistent with studies using words to cue AMs that have not found such an association^26^ and supports the conclusion that AM overgenerality is a trait marker of depression.^27^

These preliminary results could have implications for furthering management options for MDD. Several interventions targeting increasing memory specificity in patients with MDD have shown positive results,^28,29^ suggesting that improving autobiographical specificity may lead to reduced depressive symptoms. Here, we provide a method to directly increase memory specificity in a single session using simple, easily accessible odor cues that could be readily found at a grocery store. Whether this could be used to develop a novel memory-specificity training intervention is a direction for future research. Future work is also needed to understand the neural mechanisms of odor-cued AMs vs word-cued AMs, particularly to test the hypothesis that amygdala and hippocampal activity are increased in response to odor cues. If such an association is found, it could also highlight a potential mechanistic account for how neurofeedback training to increase the amygdala response to positive specific memory recall results in clinical improvements and improved memory specificity.^19^ Because odors are thought to directly target the amygdala, odor-cued memory-specificity training could be a more affordable and accessible alternative to the neurofeedback component from this intervention for ensuring the amygdala is activated during positive memory recall. It should be noted that the gustatory cortex also has direct projections to the amygdala.^30^ Therefore, taste cues could also conceivably increase AM specificity and should be the subject of future research. However, there are more barriers to implementation for taste cues, including participants’ willingness to engage with such a protocol and participant allergies, than for odor cues.

### Limitations

This study has limitations. The main limitation is that we did not include a healthy control group. This limits our ability to draw firm conclusions regarding whether the overgeneral memory phenomenon occurred in our sample. However, in the verbal cue condition, 52% of memories were specific, while in the odor cue condition, 68% of memories were specific. A 1 sample *t* test comparison with the healthy population mean of 80% specific memories suggests that our verbal cues produced fewer specific memories, consistent with the presence of an overgenerality, but that specific recall was no different from the healthy population mean when odor cues were used. However, without the addition of a healthy control sample, it cannot be ruled out that the conditions may have been somewhat different. Another limitation of our study is the relatively small sample size. While we had sufficient power to detect and can be confident in our primary outcome, larger sample studies are needed to further understand the associations of other properties of the recalled memories, including valence. A final limitation of this study is the sample of primarily White female participants; however, this is generally representative of the population of patients with a primary diagnosis of MDD.^31^

## Conclusions

These findings suggest that participants with MDD recall more specific AMs in response to odor cues than word cues. Additionally, we found that these AMs are rated more arousing and vivid upon recall, suggesting further support for the Proust phenomenon.^12^ This study suggests the potential for increasing AM specificity in individuals with MDD with the future goal of reducing depression symptoms for this population and informing a better understanding of the neural mechanisms influencing odor-based AM recall. We hope this initial study spurs larger studies in more diverse samples that include healthy control participants to further investigate and explain these associations.

## Notes

Supplement.
Data Sharing Statement
