# Supplementary material for: Recall of Autobiographical Memories Following Odor vs Verbal Cues Among Adults With Major Depressive Disorder
Source: JAMA Netw Open. 2024 Feb 13;7(2):e2355958. doi: 10.1001/jamanetworkopen.2023.55958 (PMC10865143; doi:10.1001/jamanetworkopen.2023.55958)
Supplement: Supplement. — Data Sharing Statement [file jamanetwopen-e2355958-s001.pdf]

Data Sharing Statement

Leiker. Recall of Autobiographical Memories Following Odor vs Verbal Cues Among Adults With Major Depressive Disorder. *JAMA Netw Open*. Published February 13, 2024. doi:10.1001/jamanetworkopen.2023.55958

Data
Data available: Yes
Data types: Deidentified participant data
How to access data: Data may be requested from the senior author at youngk@pitt.edu
When available: With publication

Supporting Documents
Document types: None

Additional Information
Who can access the data: Anyone requesting the data
Types of analyses: For any purpose
Mechanisms of data availability: with a signed data access agreement
